# Efficient Biorenewable Membranes in Lithium-Oxygen Batteries

**DOI:** 10.3390/polym15153182

**Published:** 2023-07-26

**Authors:** Julia Amici, Giorgio Banaudi, Mattia Longo, Matteo Gandolfo, Michael Zanon, Carlotta Francia, Silvia Bodoardo, Marco Sangermano

**Affiliations:** Department of Applied Science and Technology, Politecnico di Torino, c.so Duca degli Abruzzi 24, 10129 Torino, Italymattia.longo@polito.it (M.L.); marco.sangermano@polito.it (M.S.)

**Keywords:** lithium-oxygen battery, metallic lithium protection, photopolymerization, bio-renewable resources

## Abstract

Lithium-oxygen batteries, with their very high energy density (3500 Wh kg^−1^), could represent a real breakthrough in the envisioned strategies towards more efficient energy storage solutions for a less and less carbonated energy mix. However, the problems associated with this technology are numerous. A first one is linked to the high reactivity of the lithium metal anode, while a second one is linked to the highly oxidative environment created by the cell’s O_2_ saturation. Keeping in mind the necessity for greener materials in future energy storage solutions, in this work an innovative lithium protective membrane is prepared based on chitosan, a polysaccharide obtained from the deacetylation reaction of chitin. Chitosan was methacrylated through a simple, one-step reaction in water and then cross-linked by UV-induced radical polymerization. The obtained membranes were successively activated in liquid electrolyte and used as a lithium protection layer. The cells prepared with protected lithium were able to reach a higher full discharge capacity, and the chitosan’s ability to slow down degradation processes was verified by post-mortem analyses. Moreover, in long cycling conditions, the protected lithium cell performed more than 40 cycles at 0.1 mA cm^−2^, at a fixed capacity of 0.5 mAh cm^−2^, retaining 100% coulombic efficiency, which is more than twice the lifespan of the bare lithium cell.

## 1. Introduction

The growing need for clean energy production and storage due to global warming has prompted research into cleaner and more efficient energy storage solutions. While Li-ion secondary batteries have literally revolutionized our lives since their first commercialization in 1991, they are now reaching their theoretical energy density and therefore won’t represent a viable solution for the much-needed energy transition. A possible groundbreaking answer could arrive from the next generation of metal-oxygen batteries (MOBs), directly using atmospheric oxygen as active material [1,2]. Among those, one of the most promising technologies currently under study is the lithium–oxygen battery (LOB), thanks to its high energy density, theoretically around 3500 Wh kg^−1^ based on the formation of lithium peroxide [3,4]. This high energy density can be explained by the low metallic lithium density and low standard reduction potential (E° = −3.04 V) [5]. Despite such impressive but theoretical energy storage abilities, LOBs are still far from practical applications because of their severe side reactions, which provoke high overpotential and thus limit their cycling life [6,7]. In particular, looking at the anode side, the low cycling stability can be attributed to the intrinsic instability of lithium metal, causing dendrite nucleation and growth upon cycling, which tend to form dead lithium, continuously consume solvent to form a fresh passivation layer, and can eventually result in short circuits [8,9]. Moreover, it is worth noting that, compared to other Li-metal batteries, the issues associated with metallic lithium in LOBs are actually worsened by the presence of reactive oxygen species, leading to severe corrosion phenomena [3]. As a matter of fact, the intrinsic corrosion induced by O_2_ as well as discharge intermediates, especially the superoxide O_2_^−^ and the peroxide O_2_^2−^ radical anions in the electrolyte, leads to LOB’s inferior reversibility [10,11]. These elements indicate that an efficient anode protection strategy, helping to mitigate these issues, could represent a solution to reaching LOBs higher performances [12]. To this end, different roads have been followed, such as lithium pretreatment [10], electrolyte additives to generate in situ a stabler solid electrolyte interphase (SEI) [7,11], inorganic protective layers [13], polymer layers [8,12], or composite layers [3,6,14]. However, no definitive solution has been encountered yet, as each envisioned road has its drawbacks. For example, inorganic conductive layers are usually too fragile and expensive for practical uses, and electrolyte additives cannot control the physical strength of the artificial SEI and therefore cannot efficiently prevent dendrite formation at high current densities and capacities [15]. Regarding polymer or composite protective layers, most solutions reported in the literature use fluoropolymer-based membranes, such as Nafion [14] or polyvinylidene fluoride (PVDF) [6,8,12]. However more and more research in the battery field is looking towards the substitution of fluorinated compounds by greener solutions. Therefore, keeping in mind the objective of developing a future technology, attention should be focused not only on system performance but also on material sustainability [16]. Within the battery research area, different works have been carried out to replace existing materials with bio-renewable ones, such as binders obtained from polysaccharides [17], vegetable oils [18,19], or carbons obtained from green waste [20]. However, few of these papers concern materials for LOBs.

Herein, we focused on the second most abundant polymer in nature, after cellulose, which is chitin [21]. More precisely, chitosan, derived from the deacetylation of chitin, has been commercially manufactured from shrimp and crab shells for years and has a wide field of applications [22,23,24,25]. In addition to being a green and biorenewable polymer, medical and pharmaceutical studies have demonstrated its interesting antioxidant properties, in particular its high superoxide radical scavenging activity [22,26,27,28]. As a matter of fact, chitosan presents one amino group and two hydroxyl groups in each of its monosaccharide construction units. While the hydroxyl can react with free radicals in a typical H-abstraction reaction [29], the amino groups can react with free radicals to form additional stable macroradicals, according to the free radical theory. Therefore, the active amino and hydroxyl groups in the polysaccharide are at the origin of the scavenging ability of chitosan [30]. This peculiarity is here exploited for the first time in LOBs, where the O_2_ excess condition generally provokes the formation of excess superoxide radicals, which in turn accelerate electrolyte decomposition, thus clogging the porous electrode and impeding further O_2_ access while passivating the lithium surface. In addition, thanks to their high polarity, the same chitosan functional groups should enhance lithium salt dissociation [31,32], thus improving the Li^+^ cation mobility and reducing the concentration polarization, which is one of the reported causes of dendrite nucleation [33,34].

Therefore, in this work, chitosan molecules were methacrylated in water through a simple one-step reaction and then cross-linked by UV-induced radical polymerization. The obtained membranes were successively activated in a liquid electrolyte (LiTFSI 0.5 M in DMSO) and used as an innovative lithium protection layer. The cells prepared with protected lithium were able to perform more than 40 cycles at 0.1 mA cm^−2^, at a fixed capacity of 0.5 mAh cm^−2^, under a constant O_2_ flow of 4 mL min^−1^, at room temperature, retaining 100% coulombic efficiency. This is actually more than twice the lifespan of a similar cell containing bare metallic lithium, which failed in the same testing conditions after 19 cycles. 

## 2. Materials and Methods

### 2.1. Materials

Chitosan medium molecular weight (CH M_MW_, Mw = 190–310 KDa, 75–85% degree of N-deacetylation), chitosan low molecular weight (CH L_MW_, Mw < 100 kDa, 75–85% degree of N-deacetylation), methacrylic anhydride (MA, 94%), acetic acid (Z, 99%), 2-hydroxy-1-(4-(2-hydroxyethoxy)phenyl)-2-methylpropan-1-one (Irgacure 2959), dimethyl sulfoxide (DMSO, anhydrous ≥ 99.9%), and lithium bis(trifluoromethanelsulfonyl)imide (LiTFSI) were all purchased from Sigma–Aldrich (Milan, Italy) and used as received without further purification.

### 2.2. Synthesis of Methacrylated Chitosan

The methacrylation reaction was performed both on low- and medium molecular-weight chitosan (CH). Briefly, CH (1.5 wt%) was solubilized in a 2 wt% aqueous acetic acid solution at 50 °C. Once a homogeneous solution was obtained, MA was added dropwise until reaching a molar ratio of 20:1 (MA:CH). Afterward, the solution was left under constant stirring at 50 °C for 4h. The obtained product was dialyzed for 4 days and subsequently freeze-dried.

### 2.3. UV-Curing

Methacrylated chitosan (1.5 wt%) was solubilized in a 2 wt% aqueous solution of acetic acid. Then, 2 wt% (with respect to the chitosan mass) of Irgacure 2959 was added to the solution and solubilized. Afterward, the solution was cast on a disc-shaped silicon mold and exposed to UV light for 7 min (with an energy of 108 mW cm^−2^). The obtained membranes were peeled off, dried in air at room temperature for 15 h and, subsequently, under vacuum at 40 °C for 16h. The membranes obtained from low molecular weight methacrylated chitosan are referred to as CHMA L_MW_, and the ones from medium molecular weight are referred to as CHMA M_MW_.

### 2.4. Characterization Methods

The chemical composition of the products was evaluated through attenuated total reflectance infrared spectroscopy (ATR-FTIR). The experiments were conducted on freeze-dried samples on a Thermo Scientific Nicolet iS50 FTIR Spectrometer (Milano, Italy), equipped with a diamond crystal ATR accessory. Thirty-two ATR spectra were collected with a resolution of 4 cm^−1^ in the range of 4000–600 cm^−1^ for each sample. The spectrum of original chitosan was taken as a reference.

The photorheology tests were performed with an Anton PAAR Modular Compact Rheometer (Physica MCR 302, Graz, Austria) using a parallel plate configuration (diameter = 15 mm) with a quartz bottom glass. The gap value was set at 300 μm. The time sweep experiment was performed in the linear viscoelastic region (LVR) at a constant strain amplitude (γ) of 0.5% and a constant frequency (ω) of 5 rad s^−1^, in order to monitor the in situ gel formation by following the evolution of elastic storage modulus G’ with time. The reaction can be considered complete when the G’ plateau is reached. In these experiments, the UV lamp (LC8, Hamamatsu, Japan, with a light intensity of 28 mW cm^2^) was switched on after 30 s. All experiments were carried out at room temperature.

Thermogravimetric analysis (TGA) was used to assess thermal stability with a Netzsch TG 209 F3 (NETZSCH-Gerätebau GmbH, Verona, Italia), in N_2_, between 25 and 800 °C, with a temperature ramp of 10 °C min^−1^.

The liquid electrolyte uptake (LEU) was monitored at different time steps after removing the sample from the liquid electrolyte (0.5 M LiTFSI in DMSO) and weighting it after eliminating the liquid excess on the surface. The electrolyte uptake was calculated according to Equation (1):

(1) $$LEU=\frac{M_{e}-M_{0}}{M_{0}}\times 100,$$

where *M_0_* and *M_e_* are the weights of the membrane before and after immersion, respectively.

The interfacial stability of the prepared membranes against lithium was studied by electrochemical impedance spectroscopy (EIS) at open circuit voltage (OCV) on a versatile multichannel potentiostat (VMP-3 Biologic, Grenoble, France) through Li/Membrane/Li symmetric cells (ECC-Std test cells, EL-CELL GmbH, Hamburg, Germany). The frequency of the EIS ranged from 10^5^ Hz to 1 Hz, with an amplitude of 10 mV.

The electrochemical stability was evaluated by linear sweep voltammetry (LSV) performed at a scan rate of 0.1 mV s^−1^ from 2.5 to 5 V vs. Li^+^/Li at room temperature on a SS|Membrane|Li cell (ECC-Std test cells, EL-CELL GmbH, Hamburg, Germany).

The ionic conductivity was determined by EIS using a versatile multichannel potentiostat (VMP-3 Biologic, Grenoble, France) in the frequency range between 10^5^ Hz and 1 Hz at OCV. The prepared membranes, activated with liquid electrolyte, were sandwiched between two stainless steel blocking electrodes (ECC-Std test cells, EL-CELL GmbH, Hamburg, Germany). The assembled cells were kept in a MKF56 dynamic climatic chamber (Binder, Tuttlingen, Germany) and tested between 25 and 60 °C. The resistance of the electrolyte was given by the high-frequency intercept determined by analyzing the Nyquist plot of the impedance response. The ionic conductivity was calculated at each temperature using Equation (2):

(2) $$\sigma =\frac{l}{A}\times \frac{1}{R_{Ω}},$$

where *l* is the membrane thickness, *A* is the membrane surface area, and *R*_Ω_ is the resistance value at the high-frequency intercept.

The lithium-ion transference number (*t_Li+_*) was measured using symmetrical Li|Membrane|Li cells by a potentiostatic polarization method performed on a versatile multichannel potentiostat (VMP-3 Biologic, Grenoble, France). The lithium-ion transference number *t_Li+_* was calculated using the Bruce and Vincent model, following Equation (3) [35]:

(3) $$t_{Li^{+}}=\frac{I_{s}\times \left(\Delta V-I_{0}\cdot R_{0}\right)}{I_{0}\times \left(\Delta V-I_{s}\cdot R_{s}\right)},$$

where *I*_0_ and *I*_S_ are the initial and steady-state currents, respectively. Δ*V* is the DC potential (10 mV) applied to chronoamperometry; *R*_0_ and *R*_S_ are the interfacial impedance at initial and steady state, respectively, which are measured by EIS.

The effect of the membrane on Li plating and stripping was studied using a Li/Li symmetrical cell configuration with the corresponding membranes sandwiched in between (ECC-Std test cells, EL-CELL GmbH, Hamburg, Germany). The tests were performed at a 0.1 mA cm^−2^ current density with a fixed capacity of 0.1 mAh cm^−2^. 

For each electrochemical characterization technique mentioned before, the results were compared with those of a control cell assembled with a commercial glass fiber separator (GF, 18 mm × 0.65 mm, ECC1-01-0012-A/L, EL-CELL GmbH, Hamburg, Germany) impregnated with 200 µL of the liquid electrolyte (LE) 0.5 M LiTFSI in DMSO in place of the different protective membranes. In the corresponding figures, this control cell is referred to as GF + LE.

For full-cell testing, discs with an area of 2.54 cm^2^ were cut from a commercial carbon paper gas diffusion layer (GDL-24BC, SIGRACET SGL Technologies, Meitingen, Germany), dried in vacuum at 120 °C for 6 h, and used as a cathode. The GF separator, impregnated with 200 µL of 0.5 M LiTFSI in DMSO, was used as the electrolyte. A bare Li disc (18 mm × 0.2 mm, Chemetall s.r.l., Giussano, Italy) was used as the anode for the “Bare Li” cells, while membrane-protected Li discs were used in the “CHMA L_MW_ protected Li” and “CHMA M_MW_ protected Li” cells. The cells were assembled in an Ar-filled glove box (Mbraun Labstar Labstar, H_2_O and O_2_ content <1 ppm) using an ECC-Air electrochemical cell design (EL-CELL GmbH, Hamburg, Germany). The cells were galvanostatically cycled on an Arbin BT-2000 battery tester (College Station, TX, USA) at room temperature. During measurements, pure O_2_ at a flow rate of 4.0 mL min^−1^, was constantly fluxed. Prior to each test, cells rested under oxygen flow for 6 h at OCV.

In order to investigate the morphology and nature of discharge and charge reaction products on the cathode surface, some cells were disassembled in a glovebox, and the corresponding cathodes were extracted, washed with DMSO, and characterized by field-emission scanning-electron microscopy (FESEM, ZEISS Supra 40, Oberkochen, Germany), as well as X-ray diffraction (XRD) analysis. This last one was performed on a high-resolution Philips X’pert MPD powder diffractometer (Philips, Amsterdam, The Netherlands), equipped with Cu Kα radiation (V = 40 kV, I = 30 mA) and a curved graphite secondary monochromator. The diffraction profiles were collected in the 2Ѳ range between 15 and 65, with an acquisition step of 0.018° and a time per step of 10 s, using a solid-state PIXcel-1D detector with 255 active channels.

## 3. Results and discussion

### 3.1. Physico-Chemical Characterizations

**Scheme 1:**
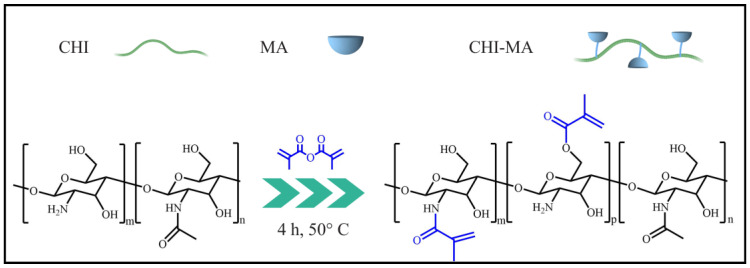
Schematic representation of chitosan methacrylation.

The methacrylation reaction of medium- and low-molecular-weight chitosan, illustrated in Scheme 1, was performed following previous works reported in the literature [36,37].

**Figure 1:**
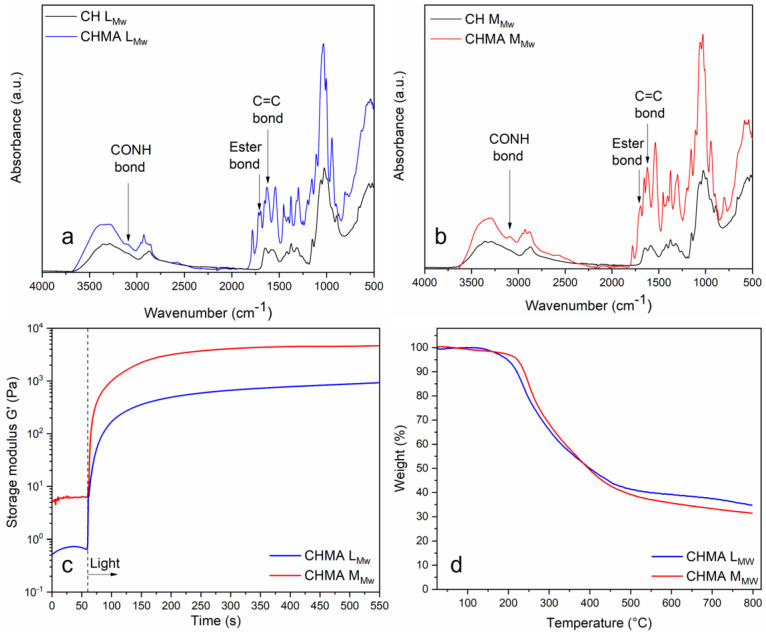
FTIR spectra of low molecular weight chitosan before and after methacrylation (**a**) and medium molecular weight chitosan before and after methacrylation (**b**); Photo-rheology curves for formulations containing low and medium molecular weight methacrylated chitosan (**c**); TGA traces for membranes obtained from low and medium molecular weight methacrylated chitosan (**d**).

ATR-FTIR spectroscopy was used to verify the occurrence of the methacrylation reaction. The corresponding spectra are reported in Figure 1a,b. In both cases, the peak at 1620 cm^−1^, corresponding to C=C stretching, confirms the successful methacrylation reaction of chitosan. More precisely, on the chitosan molecule, this reaction can happen both on the hydroxyl and the amine groups. This is indeed verified, for both samples, by the presence of ester bonds (peak at 1710 cm^−1^) and CO-NH bonds (peak at 3091 cm^−1^) [38,39].

Once it was confirmed that chitosan was successfully methacrylated, it was possible to obtain chitosan membranes through photo-initiated radical polymerization. To this end, as a first step, photo-rheology was performed to assess the curing time as well as the storage modulus of the polymers obtained from medium- and low-molecular-weight methacrylated chitosan solubilized in water. To do so, 2 wt % of chitosan was dispersed in an aqueous solution of acetic acid (2%) before adding the photoinitiator (2 wt% with respect to chitosan). The measurement was performed, monitoring the storage modulus evolution during irradiation time. Indeed, for both samples, a very sharp initial G’ increase can be observed from the UV irradiation (Figure 1c), showing the fast kinetics of the UV-Curing reaction [40]. In both cases, a plateau was reached after approximately 350 s, but the storage modulus reached by the medium molecular weight sample (CHMA M_MW_) was more than 4 times higher than the one reached by the low molecular weight sample (CHMA L_MW_), with 4.7 kPa and 0.93 kPa, respectively, indicating a probably higher crosslinking density than the first one.

After assessing the very high reactivity of the methacrylated chitosan under UV irradiation, similar precursor solutions were cast into circular silicon molds and exposed to UV lights for 7 min. The obtained membranes are transparent, self-standing, and flexible, as can be seen in Figure S1, and their average thickness is 200 µm. Thermal resistance was studied through thermogravimetric analysis; the results are reported in Figure 1d. Both samples have quite similar behavior, with one main weight loss between 200 and 500 °C caused by the breaking of chitosan chains and the formation of volatile compounds [41]. The degradation temperature is slightly higher for UV-Cured samples obtained from medium molecular weight chitosan compared to the samples obtained from low molecular weight chitosan. Interestingly, no noticeable weight loss occurs between 25 and 200 °C, thus confirming the absence of adsorbed water in the samples and therefore the efficiency of the drying process. This is indeed a critical parameter, as chitosan is quite hydrophilic and metallic lithium reacts strongly with water.

### 3.2. Electrochemical Characterizations 

**Figure 2:**
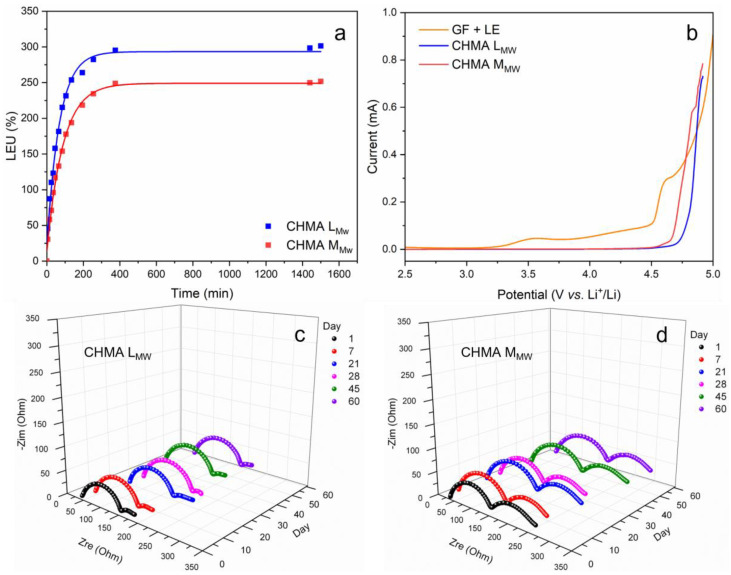
Liquid electrolyte uptakes for the membranes CHMA L_MW_ and CHMA M_MW_ (**a**); LSV profiles of CHMA L_MW_ and CHMA M_MW_ compared to a commercial glass fiber separator impregnated with liquid electrolyte (LiTFSI 0.5 M in DMSO) (**b**) Interfacial stability was assessed through EIS at OCV on a Li|CHMA L_MW_|Li cell (**c**) and on a Li|CHMA M_MW_|Li cell (**d**) at room temperature.

For these samples to be used as metallic lithium protection, in addition to being free of water traces, they must also allow for efficient and uniform Li ion conduction. To this end, the obtained membranes were immersed in a commercial liquid electrolyte (0.5 M LiTFSI in DMSO), and the liquid electrolyte uptake (LEU) was assessed as a function of the immersion time. The obtained results are reported in Figure 2a. Both samples present a quick uptake in the first 200 min of the test, illustrating the good compatibility between the polymer matrix and the selected liquid electrolyte. The swelling process is quicker and more efficient, reaching 300% of LEU for the membrane obtained from low molecular weight chitosan and 250% for the one obtained from medium molecular weight chitosan. This is in good agreement with the results obtained from the photo-rheology test, where the medium-molecular-weight methacrylated chitosan showed a higher modulus upon irradiation, probably attributable to a higher crosslinking density. As a matter of fact, the lower crosslinking density of the low molecular weight chitosan implies a major freedom of the polymer chains that can easily create free space to accommodate higher amounts of liquid electrolyte.

To further verify the interfacial stability between metallic lithium and the activated membranes, symmetric cells were assembled by sandwiching them between two Li foils. Such cells were left at OCV and room temperature, and their impedances were checked regularly through EIS for 2 months. The obtained results are reported in Figure 2c,d and the corresponding equivalent circuit diagram is in Figure S2. For both cells, the impedance spectra show a combination of two resistive processes, observable as two semi-circles with different diameters. The diameter of the semi-circle at higher frequencies represents the SEI resistance (R_SEI_), while the diameter of the semi-circle at lower frequencies represents the charge transfer resistance (R_CT_) [42]. The total interfacial resistance, which is the sum of R_SEI_ and R_CT_ [32], initially increases for both cells, indicating the growth of the SEI layer on both lithium interfaces, before interfacial stabilization is reached, both for medium and low molecular weight chitosan membranes [43]. Interestingly, though, the EIS profiles are quite different between the two cells, and while the R_SEI_ values are quite similar, around 150 Ω, the R_CT_ value is much higher for CHMA M_MW_ compared to CHMA L_MW_, indicating a more difficult charge transfer reaction in the second case. One explanation can be linked to the mechanical properties of the two membranes; indeed, as demonstrated by the photo-rheology test (see Figure 1c), the membrane obtained from the UV curing of low molecular weight chitosan is softer than the other one, thus allowing for a much better contact with the surface of lithium and, therefore, a decreased interfacial resistance, resulting in a minor R_CT_.

In addition to good interfacial stability at OCV, it is important to assess the membrane’s resistance to oxidative degradation at higher potential. To this end, LSV was performed between 2.5 and 5 V vs. Li^+^/Li at a scan rate of 0.1 mV s^−1^. As can be seen in Figure 2b, the DMSO-based liquid electrolyte starts oxidizing at a relatively low potential (around 3.25 V vs. Li^+^/Li) when it is supported by a commercial glass fiber separator (GF + LE), even if this phenomenon is quite limited as the corresponding residual current is very small [44]. Interestingly, the entrapment of the same liquid electrolyte in the polymer matrix seems to slow down the process and protect it from oxidation up to potentials higher than 4.5 V vs. Li^+^/Li, probably thanks to the strong interaction between the polymer matrix and the absorbed electrolyte [32].

**Figure 3:**
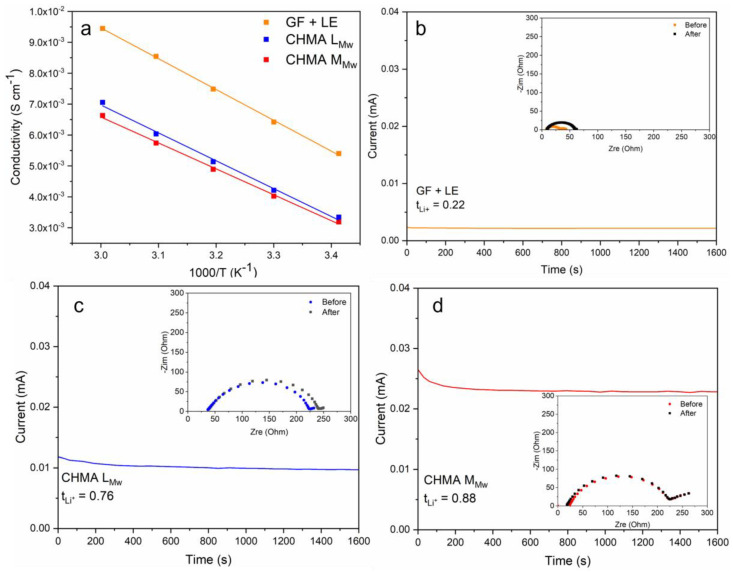
Ionic conductivity vs. temperature plot for the GPEs CHMA L_MW_, CHMA M_MW_, and a glass fiber separator impregnated with liquid electrolyte (LiTFSI 0.5 M in DMSO) (**a**); current-time profile of a Li|GF+ LE|Li cell (**b**); a Li|CHMA L_MW_|Li cell (**c**); and a Li|CHMA M_MW_|Li cell (**d**). The insets show the EIS spectra before and after polarization.

Once the stability of the protective membranes was assessed, both at OCV and at higher potentials, and thanks to their excellent swelling abilities, it was possible to assess their ionic conductivities through EIS, performed on symmetric cells (SS|Membrane|SS), at different temperatures. The results obtained with CHMA L_MW_ and CHMA M_MW_ were compared to a symmetric cell assembled with a commercial glass fiber separator impregnated with the same liquid electrolyte (0.5 M LiTFSI in DMSO). As can be seen in Figure 3a, while the membrane conductivities are below those of GF + LE, the order of magnitude is still the same and, in both cases, superior to 1 mS cm^−1^ at room temperature, with 1.9 mS cm^−1^ for the medium molecular weight sample and 2.2 mS cm^−1^ for the low molecular weight one. Such a small difference could be explained once more by the higher crosslinking density of the medium molecular weight sample, which restricted the segmental motion of the polymer chains and therefore the Li ion motion.

While the previous measurement assessed the global ionic conductivity, including both anions and cations movements, the lithium transference number allows us to specifically understand the fraction of electric current transported by the cation Li^+^. This parameter is fundamental for an efficient metallic lithium protection layer; indeed, the closer *t_Li+_* is to a unitary value, the lower the concentration gradient across the layer will be, thus reducing the anion accumulation at the interface with the electrodes [45]. Such charge accumulation near the lithium surface has been widely demonstrated in the literature to be one of the primary causes of dendrite nucleation and growth [33,34]. The results reported in Figure 3b–d show a *t_Li+_* number four times higher for the membrane obtained from medium molecular weight chitosan compared to the liquid electrolyte on a glass fiber separator, while it is slightly lower for the CHMA L_MW_ membrane. Such impressive results can be explained by the large presence, on the chitosan molecules, of highly polar hydroxyl and amine groups, which enhance lithium salt dissociation [31,32], as well as by the strong ability of C=O groups from the methacrylic functionalities to withdraw electrons and restrict the mobility of the anion [43,46].

**Figure 4:**
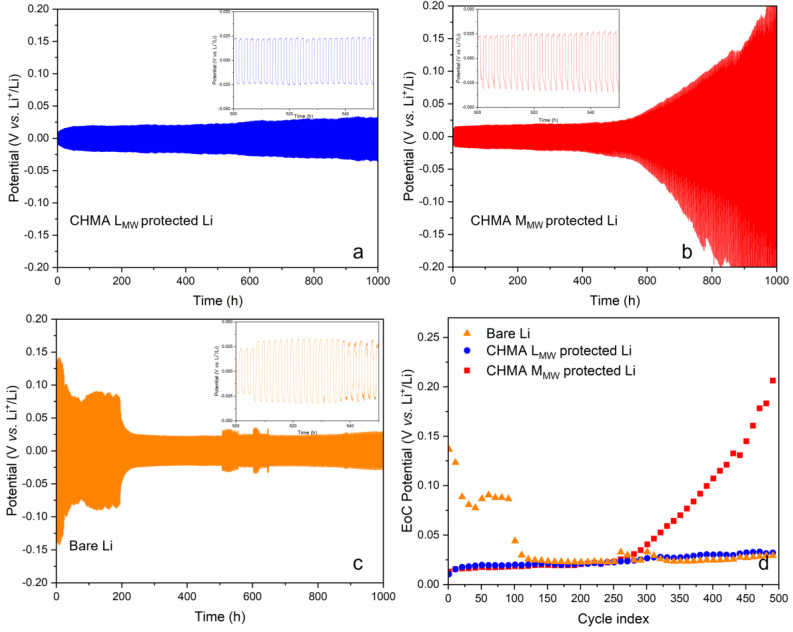
Galvanostatic lithium plating and stripping at a current density of 0.1 mA cm^−2^ and the fixed capacity of 0.1 mAh cm^−2^, at room temperature, on a Li|CHMA L_MW_|Li cell (**a**), a Li|CHMA M_MW_|Li cell (**b**), and a Li|GF+ LE|Li cell (**c**), end of charge potential for the previous three symmetric cells (**d**).

Lithium-ion transport kinetics upon the protective membranes, as well as the interfacial stability upon cycling, were further investigated by galvanostatic Li plating and stripping on symmetric Li|membrane|Li cells at a current density of 0.1 mA cm^−2^ with a limited capacity of 0.1 mAh cm^−2^. The obtained results were compared to those of a symmetric cell containing a glass fiber separator soaked with the same liquid electrolyte. As can be seen in Figure 4a–c, the obtained profiles differ quite a lot between the chitosan-based membranes and the glass fiber cells (Figure 4c). Looking in particular at the “End of Charge” potential (Figure 4d), it is possible to see that this last one presents, initially, an important overvoltage that is then able to stabilize after more than 200h. On the contrary, the overvoltage of the CHMA L_MW_ and CHMA M_MW_ is quite small right from the beginning thanks to the uniform Li ion transport through the GPEs as well as the enhanced interfacial stability towards lithium metal [42]. After 300 cycles, the stability of the CHMA M_MW_ membrane is inferior compared to the CHMA L_MW_ one. This is probably linked to the higher crosslinking density of the first one, resulting in poorer interfacial contact and, upon cycling, the formation of a less stable and more resistive interface. This is further corroborated by the results of the interfacial stability characterization (see Figure 2c,d), where it was seen that CHMA M_MW_ had a far higher R_ct_ compared to CHMA L_MW_.

Focusing on the potential profiles between 500 and 550 h (insets in Figure 4a–c), while the profiles for the CHMA L_MW_ protected Li have a perfectly regular and reproducible shape, with a symmetric overpotential between −0.025 and +0.025 V, confirming the formation of a stable interface with lithium, the profiles for the CHMA M_MW_ protected Li are less symmetrical, with a pointier shape in the negative potentials. This is in line with the higher R_ct_ value between this membrane and lithium, making the plating and stripping of Li more difficult across the interface [47,48]. Interestingly, such a phenomenon seems to be worse for one interface relative to the other, explaining the non-symmetrical profile. Last but not least, the profiles of the bare Li cell are much less regular, with a fluctuating over-voltage and, for the last cycles reported, a noisy behavior. This confirms the formation of a less stable interface and a more resistive SEI with the probable presence of dead Li, hindering the Li plating and stripping processes [49].

### 3.3. Lithium-Oxygen Cells Performance

**Figure 5:**
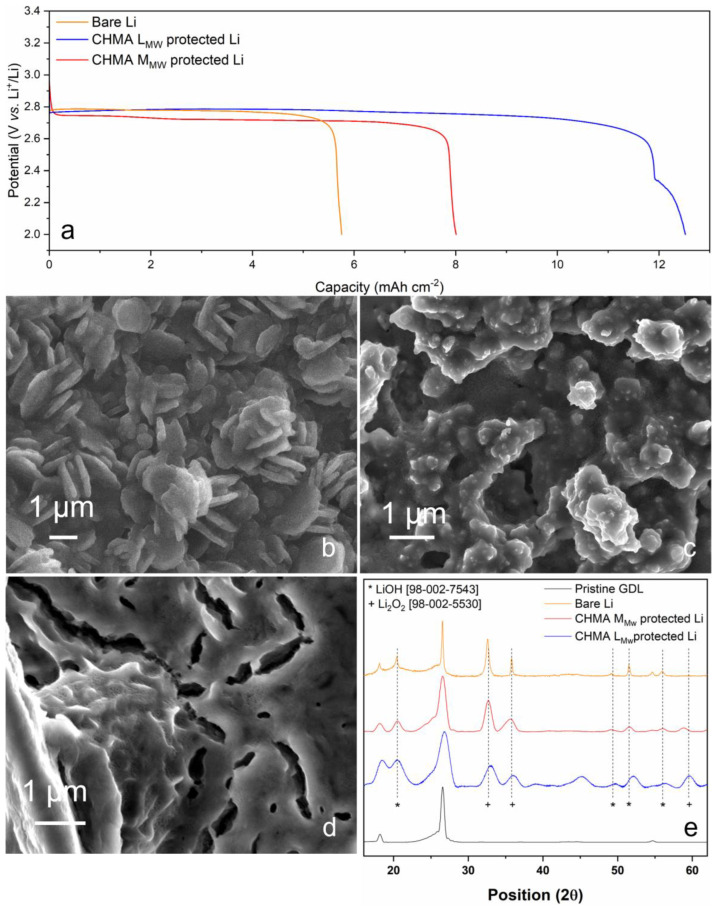
Full discharge voltage profiles at 0.1 mA cm^−2^ down to 2.0 V (**a**), Cathode post-full discharge FESEM micrograph for the bare lithium cell (**b**), CHMA L_MW_ protected lithium cell (**c**), CHMA M_MW_ protected lithium cell (**d**), corresponding XRD spectra compared to the one of pristine cathode (**e**).

Galvanostatic discharge tests were performed, from OCV down to 2.0 V vs. Li^+^/Li, at a current density of 0.1 mA cm^−2^, to assess the full discharge capacity of a cell containing bare lithium as an anode and two cells containing protected lithium (namely with CHMA L_MW_ and CHMA M_MW_). The cells were assembled with a commercial gas diffusion layer as a cathode, a glass fiber separator impregnated with liquid electrolyte (0.5 M LiTFSI in DMSO), and either bare Li metal or protected Li metal as an anode. The cells were kept under a constant O_2_ flow of 4 mL min^−1^. The results reported on Figure 5a show that both cells with protected lithium were able to discharge for a longer time compared to the cell with bare lithium, reaching areal capacities of 8 mAh cm^−2^ for the CHMA M_MW_ protected Li and 12.5 mAh cm^−2^ for the CHMA L_MW_ protected Li, which is more than twice the areal capacity achieved by bare Li with 5.8 mAh cm^−2^. Previous works demonstrated that degradation products generated by DMSO decomposition, caused by prolonged contact with Li_2_O_2_, in particular in long discharge conditions, could be found both on the anode (in the SEI layer) and on the cathode surfaces [50,51,52]. This usually results in an abrupt potential drop, ending the discharge process. Here it is possible to observe how the studied protecting membranes are indeed able to efficiently slow down such phenomena. While the characterization of the Li metal anode is quite difficult due to its high reactivity, the characterization of the cathode surface is quite easier. Therefore, to verify our previous assessment, the three cells were disassembled in a glovebox after a full discharge, and their surface composition and morphology were studied by XRD and FESEM, respectively. As can be seen from the FESEM micrographs reported in Figure 5b–d, the morphologies of the three cathode surfaces are quite different. While for the bare Li cell large crystals are clearly visible (Figure 5b), for the two Li protected cells the discharge products assume a more “film-like” morphology (Figure 5c,d), with an absence of large crystalline structures, particularly in the case of the CHMA L_MW_ cell (Figure 5c). The XRD spectra of the three cathodes (Figure 5e) show similar peaks, corresponding to Li_2_O_2_, the typical discharge product of Li-O_2_ cells, and LiOH, confirming our previous hypothesis regarding DMSO decomposition. The real difference between the three spectra regards the peaks shapes; while the peaks of the bare Li cell cathode are quite narrow, the ones of the two protected Li cells are wider, which could actually confirm the presence of much smaller crystals with a reduced degree of crystallinity [53], confirming what can be observed through FESEM. Interestingly, for the CHMA M_MW_-protected Li cell, the discharge products layer on the cathode surface seems much thicker (Figure 5d), while at the same time the discharge plateau is slightly lower than for the other two cells (Figure 5a), denoting a higher cell polarization. This could be explained both by the higher R_ct_ previously assessed and by the higher cathode passivation and clogging observed through FESEM. For these reasons, CHMA M_MW_ was not further characterized, and attention was focused exclusively on the CHMA L_MW_ protective layer.

In order to assess the reversibility of such discharge products, the bare Li and the CHMA L_MW_ protected Li cells were submitted to a full recharge process, meaning that after the previously described discharge, the cells were recharged at 0.1 mA cm^−2^ up to 4.5 V vs. Li^+^/Li. The corresponding voltage profiles are reported in Figure S3b. After the potential cutoff was reached, the cells were disassembled in a glovebox, and the cathode surface morphologies were checked again by FESEM (Figure S3c,d). Comparing the recharged cathode morphologies to the surface of a pristine GDL (Figure S3a), it is possible to observe that the cathode from the CHMA L_MW_ cell recovers its initial appearance, showing no residual discharge products, while the cathode from the bare Li cell is covered by residual degradation products, thus confirming the higher reversibility of the cell containing a protected anode. Indeed, it was previously reported in the literature that the “film-like” discharge product morphology has the advantage of a higher contact area between the surface of the cathode and the discharge products, enhancing the transfer of electrons and therefore the reversibility of such products [54,55,56,57,58].

**Figure 6:**
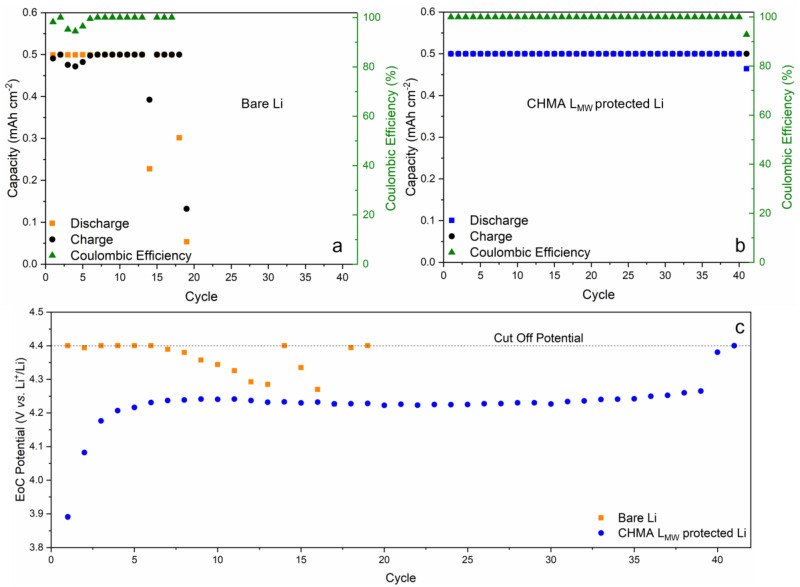
Charge/discharge capacities and coulombic efficiency (0.1 mA cm^−2^, at a fixed capacity of 0.5 mAh cm^−2^, between 2.2 and 4.4 V) for the bare lithium cell (**a**) and the CHMA LMW protected lithium cell (**b**), corresponding end of charge (EoC) potentials as a function of the cycle number (**c**).

In order to verify such assessment over long cycling conditions, a bare lithium cell and a CHMA L_MW_ protected lithium cell were assembled as reported before and cycled at 0.1 mA cm^−2^, at a limited capacity of 0.5 mAh cm^−2^ between 2.2 and 4.4 V vs. Li^+^/Li, under a constant oxygen flow of 4 mL min^−1^. Observing the charge/discharge capacities in function of the cycle number, it is possible to see that the CHMA L_MW_-protected lithium cell is able to perform more than 40 cycles with a 100% coulombic efficiency (Figure 6b), while the bare Li cell fails after 19 cycles and presents a much more fluctuating coulombic efficiency (Figure 6a).

The cycling profiles of the two cells are reported in Figure S4. While the profiles of the cell containing unprotected Li seem to present two recharge plateaus, one at 3.35 V and one around 4.0 V (Figure S4a), from the first cycle, these are not clearly visible for the cell with protected Li, where the overvoltage is overall smaller (Figure S4b). The two plateaus could actually be attributable to Li_2_O_2_ reduction and parasitic product reduction, in particular Li_2_CO_3_ [59], respectively. To better understand this overvoltage and its influence on cycling performances, Figure 6c reports the end of charge voltage in function of the cycle number, allowing one to understand if the recharge step ended because of the time limit, therefore reaching the fixed capacity value, or because of the potential limit, thus ending the recharge because of the cell polarization before reaching the fixed capacity. For the bare Li cell, it is interesting to note that for cycles 1, 3, 4, and 5, the potential cut-off is reached before the fixed capacity is completed, indicating a non-negligible cell polarization right from the beginning, as already observed in the lithium plating and striping test (Figure 4c,d). This is confirmed by Coulombic efficiency values inferior to 100% for the corresponding cycles (Figure 6a). Successively, the cell polarization decreases, probably thanks to the formation of a stabler interface with lithium, and the fixed capacity is attained while the end of charge potential (EoCP) remains smaller. Finally, after cycle 18, the cut-off voltage is reached again, and the cell fails after cycle 19th. For the CHMA L_MW_-protected Li cell, the EoCP of the first cycle is quite small, then it increases for the following 5 cycles before reaching a stable value of around 4.25 V vs. Li^+^/Li maintained up to cycle 39, demonstrating the stability of the interface between the protective membrane and the lithium. On cycle 40, the EoCP rises to 4.35 V, and on cycle 41, the cut-off potential is reached before the fixed capacity. Another difference between the two cells behavior is that, regarding the bare Li cell, the increased polarization in recharge corresponds to a failing in discharge from cycle 18th, which might be explained by the cathode pores clogging due to the accumulation of irreversible parasitic products and blocking the O_2_ flow. This phenomenon is not verified in the protected lithium cell, where the fixed capacity is always reached in discharge, even for cycles 40 and 41, indicating that, even with a lower coulombic efficiency, this cell could still cycle. In other words, the cathode failing is probably the reason for the impaired discharge process in the bare lithium cell, suggesting, as already observed in other studies, that efficient anode protection has a great impact on the cathode reactions as well [3].

A plausible hypothesis to explain the beneficial role of chitosan-based membranes on lithium metal protection in Li-O_2_ cells could be found in medical and pharmaceutical research. Indeed, different groups [60] studied the antioxidant properties of chitosan and its free radical scavenging ability. Interestingly, R. Xing et al. [61] investigated the correlation between chitosan molecular weight and its superoxide and hydroxyl radical scavenging ability, demonstrating that low molecular weight had stronger scavenging effects. Therefore, one proposed explanation could be that the CHMA L_MW_ membrane is able to scavenge the excess superoxide radicals formed during the discharge process, thus slowing down the associated degradation mechanisms such as electrolyte and cathodic carbon degradation. As a matter of fact, the bare Li cell presents the Li_2_CO_3_ recharge plateau from the first cycle, probably because of carbon/electrolyte degradation from the first cycle on. Such a second plateau remains much less obvious in the protected Li cell, thus corroborating our hypothesis.

## 4. Conclusions

In this work, a natural polymer, chitosan, was selected as a building block for a new lithium metal protection membrane designed for the highly oxidative conditions linked to the lithium-oxygen battery. To the best of our knowledge, this is the first time that chitosan’s antioxidant properties have been exploited to protect lithium metal in lithium-oxygen batteries. In particular, a low-molecular-weight and a medium-molecular-weight chitosan were methacrylated in a one-step reaction in water. Successively, the methacrylated chitosans were cross-linked via a fast, cheap, and green photopolymerization process, obtaining CHMA L_MW_ and CHMA M_MW_ membranes. Photo-rheological characterization indicated that the CHMA M_MW_ membrane reached a higher storage modulus, probably explained by a higher cross-linking density. Both membranes possess high ionic conductivities and Li transference numbers at room temperature. However, the effects of the different mechanical properties were seen on the interfacial stability test, where the R_ct_ of CHMA M_MW_ in a symmetric Li|Li cell was higher than the one of CHMA L_MW_, probably due to poorer interfacial contact with metallic lithium. This also resulted in poorer lithium plating and stripping stability, with a much higher polarization after 300h as well as a lower full discharge capacity in full cell configuration. Focusing therefore on the CHMA L_MW_ protective layer, its ability to slow down different degradation phenomena was verified, first by cathode post-mortem analysis, after full discharge and full recharge testing, allowing to assess the formation of more reversible discharge products. Secondly, it was verified in long cycling conditions where the lithium-protected cell demonstrated twice the lifespan of the bare Li one. Therefore, chitosan antioxidant properties, summed up with enhanced Li salt dissociation and thus better Li ion transport kinetics, effectively allow for interface stabilization with the anode as well as reduced degradation processes and therefore longer cycling life.

## Data Availability

Data will be made available upon request to the corresponding author.

## Supplementary Materials

The following supporting information can be downloaded at: https://www.mdpi.com/article/10.3390/polym15153182/s1. Figure S1: Pictures of CHMA L_MW_ (a) and CHMA M_MW_ (b); Figure S2: Equivalent circuit diagram; Figure S3: Pristine cathode FESEM micrograph (a), full discharge and full recharge voltage profiles (0.1 mA cm^−2^, down to 2.0 V and up to 4.5V) for the bare lithium and the CHMA L_MW_ protected lithium cell (b), cathode post full recharge FESEM micrograph for the bare lithium cell (c), and the CHMA L_MW_ protected lithium cell (d); Figure S4: Cycling profiles (0.1 mA cm^−2^, at a fixed capacity of 0.5 mAh cm^−2^, between 2.2 and 4.4 V) for the bare lithium cell (a) and the CHMA L_MW_ protected lithium cell (b).
