# Supplementary material for: Efficient Biorenewable Membranes in Lithium-Oxygen Batteries
Source: Polymers (Basel). 2023 Jul 26;15(15):3182. doi: 10.3390/polym15153182 (PMC10420843; doi:10.3390/polym15153182)
Supplement: Supplementary file 1 [file polymers-15-03182-s001.zip › polymers-2517611-supplementary.pdf]

Supporting Information

Efficient Biorenewable Membranes in Lithium-Oxygen Batteries

Julia Amici *, Giorgio Banaudi, Mattia Longo, Matteo Gandolfo, Michael Zanon, Carlotta Francia, Silvia Bodoardo and Marco Sangermano

Department of Applied Science and Technology, Politecnico di Torino, c.so Duca degli Abruzzi 24, 10129 Torino, Italy
* Correspondence: julia.amici@polito.it

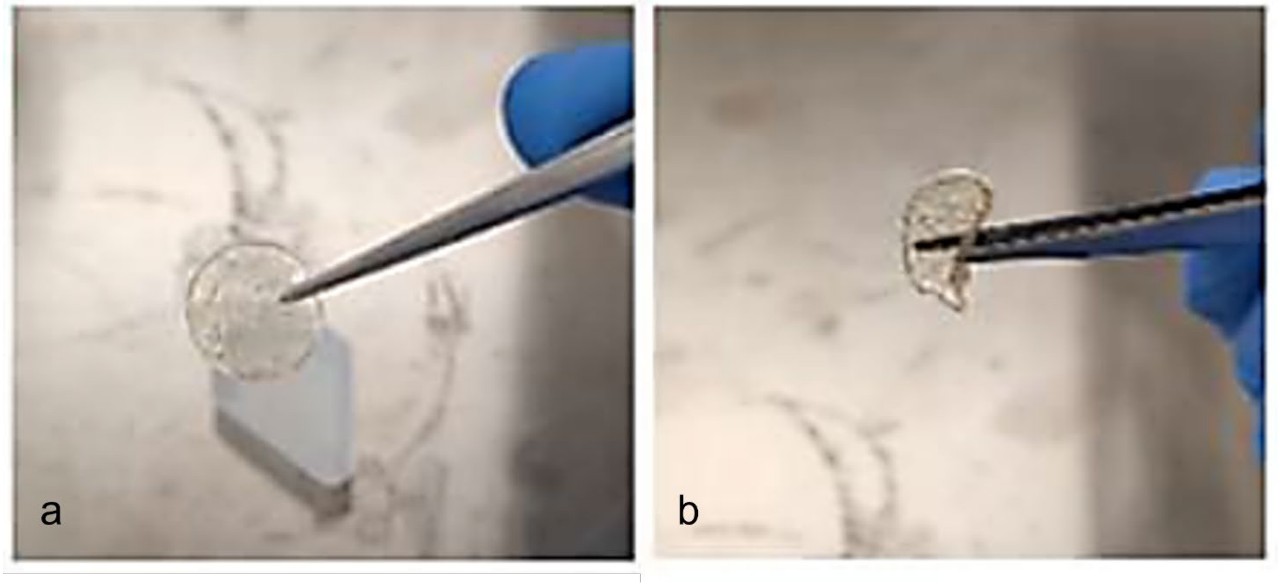

Figure S1. Pictures of CHMA L_{MW} (a) and CHMA M_{MW} (b).

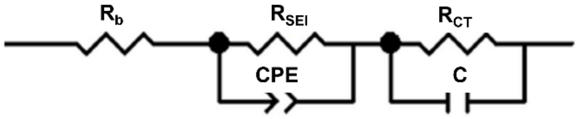

Figure S2. Equivalent circuit diagram.

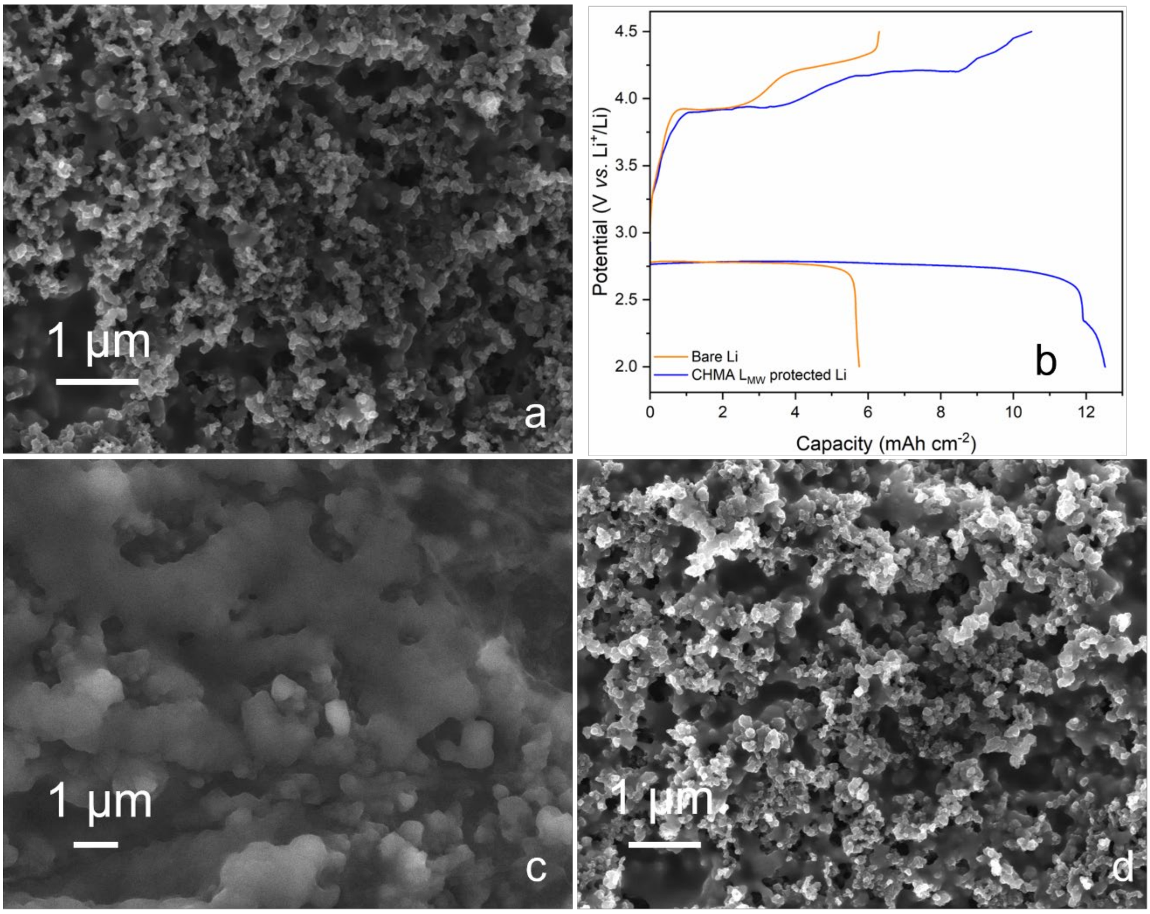

Figure S3. Pristine cathode FESEM micrograph (a), full discharge and full recharge voltage profiles (0.1 mA cm^{-2}, down to 2.0 V and up to 4.5V) for the bare lithium and the CHMA L$_{MW}$ protected lithium cell (b), cathode post full recharge FESEM micrograph for the bare lithium cell (c), and the CHMA L$_{MW}$ protected lithium cell (d).

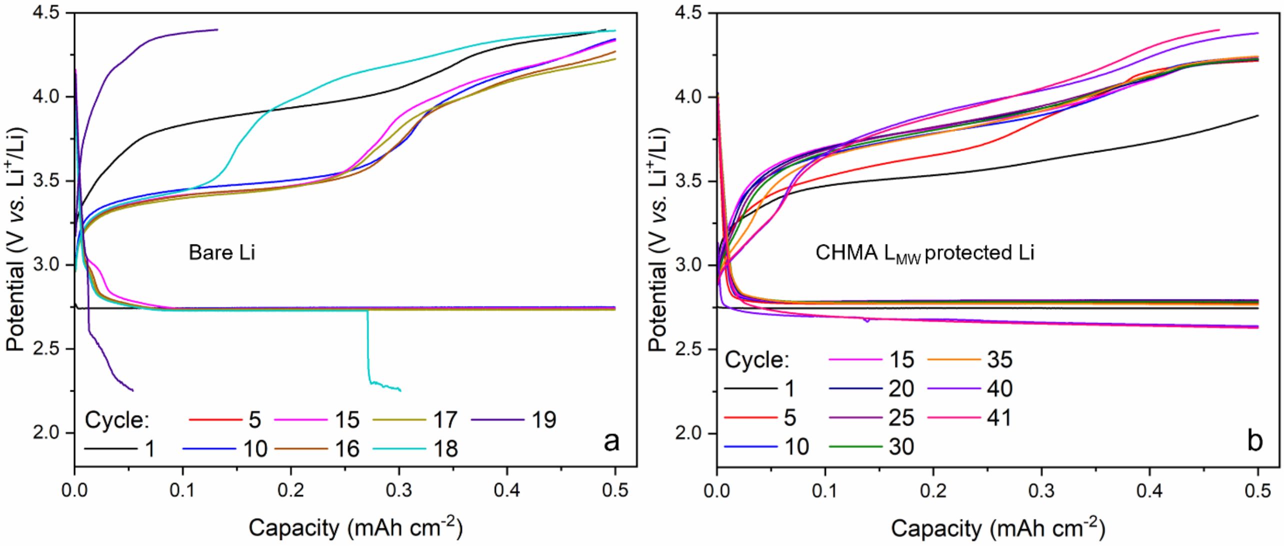

Figure S4. Cycling profiles (0.1 mA cm^{-2}, at a fixed capacity of 0.5 mAh cm^{-2}, between 2.2 and 4.4 V) for the bare lithium cell (a) and the CHMA L$_{MW}$ protected lithium cell (b).
